# Impact and process evaluation of a primary-school *Food Education and Sustainability Training* (FEAST) program in 10-12-year-old children in Australia: pragmatic cluster non-randomized controlled trial

**DOI:** 10.1186/s12889-024-18079-8

**Published:** 2024-03-01

**Authors:** F. Karpouzis, R. Lindberg, A. Walsh, S. Shah, G. Abbott, K. Ball

**Affiliations:** 1 Institute for Physical Activity and Nutrition, School of Exercise and Nutrition Sciences, Deakin University, Melbourne, VIC Australia; 2 School of Behavioural and Health Sciences, Australian Catholic University, Melbourne, VIC Australia; 3 Faculty of Medicine and Health, University of Sydney, Sydney, NSW Australia; 4 Rose Bay Nth, Australia PO Box 2108, NSW 2030

**Keywords:** School, Children, Food, Nutrition, Education, Sustainability, Fruit, Vegetable, Cluster non-randomized controlled trial, Impact evaluation, Process evaluation

## Abstract

**Background:**

Environmentally sustainable food initiatives accompanying nutrition education, such as the *Food Education and Sustainability Training* (FEAST) program, have gained traction in school settings. The aim of this trial was to conduct an impact and process evaluation of FEAST, to evaluate its effect on children’s fruit and vegetable (F&V) intakes, and secondary outcomes: F&V variety consumed, nutrition knowledge, food preparation/cooking skills, self-efficacy and behaviours, food waste knowledge and behaviours, and food production knowledge.

**Methods:**

FEAST was a 10-week curriculum-aligned program, designed to educate children about healthy eating, food waste, and sustainability, while teaching cooking skills. It was implemented by classroom teachers, face-to-face and online, during COVID-19 school closures, in Australia in 2021. A custom designed survey was used to collect baseline and post-intervention data from students. Generalised linear mixed models (GLMM) estimated group differences in pre-post changes for primary and secondary outcomes. Surveys were also administered to students and teachers to evaluate intervention implementation.

**Results:**

Twenty schools participated and self-selected to be either intervention schools (*n* = 10) or wait-list control (WLC) schools (*n* = 10). A total of 977, 5th and 6th grade children participated in the trial with a mean age of 11.1 years (SD ± 0.7). The FEAST intervention, compared to WLC, did not result in significant increases in primary outcomes nor secondary outcomes. The process evaluation revealed FEAST was well-received by students and teachers, but COVID-19 school closures hindered implementation fidelity with a less intense program delivered under the constraints of pandemic lockdowns.

**Conclusions:**

This is the first cluster non-randomized controlled trial designed to independently evaluate FEAST in the primary-school setting. No evidence was found for improved F&V intakes in children, nor secondary outcomes. However, the positive process evaluation results suggest that further trials of the program are warranted. If implemented as originally designed (pre-pandemic), with increased duration and complemented by supporting school policies, such programs have the potential to improve children’s daily F&V intakes, cooking skills and food waste behaviours. This would support the Australian curriculum and contribute to: health promotion within schools and sustainable schools initiatives, the national agenda to reduce food waste and sustainable development goals.

**Australian and New Zealand Clinical Trials Registry:**

[ACTRN12620001347954]- Registered prospectively on 14/12/2020.

**Supplementary Information:**

The online version contains supplementary material available at 10.1186/s12889-024-18079-8.

## Background

Globally, children are not meeting the recommended daily intake of five serves of fruits and vegetables (F&V) combined [1–4]. In Australia, the most recent National Health Survey reported that 91.5% of children are not meeting their recommended intakes of two serves of fruit and five serves of vegetables/day [5]. Low F&V consumption in childhood tracks into adolescence and adulthood and has been linked to the development of non-communicable diseases (NCDs) such as overweight and obesity, type-2 diabetes, cardiovascular disease, stroke and a variety of cancers [6–10].

Unhealthy diets, that include low F&V consumption, have contributed globally to the burden of diet-related NCDs as well as environmental degradation [11]. Therefore, changes in dietary intakes that incorporate increased plant-based foods and decreased highly processed, energy-dense foods are needed to reduce the diet-related burden of disease and the environmental impact of the food system [11, 12].

Numerous systematic reviews have assessed the effectiveness of school-based interventions designed to increase F&V consumption [13–18] or promote a healthy diet (including F&V intakes) [19–21]. Reviews have revealed inconsistent findings [20, 22, 23], generally reporting only small to moderate increases in combined F&V intakes [15–17, 24]. Most studies attribute increases to fruit intake alone while only increasing vegetable intakes marginally [13, 22], if at all [16]. Accordingly, there remains a need to develop different strategies to promote F&V consumption more effectively, among this cohort [16, 21, 25–27].

Environmentally sustainable food initiatives accompanying nutrition education and health promotion programs have gained traction in the school setting [28]. Understanding the environmental origins of food is increasingly recognized as a key component of food literacy and is important for promoting healthy eating [11]. In Australia and internationally, there is an urgent need to identify and implement strategies to promote and support healthy and sustainable diets, which are predominantly plant-based [29–32].

One such program was designed by OzHarvest, one of Australia’s leading not-for-profit food rescue organizations, called *Food Education and Sustainability Training* (FEAST) [33]. The FEAST program aims to educate and upskill children in healthy eating, sustainability and food waste minimisation strategies, while teaching them to cook, with a focus on increasing F&V consumption - the two most wasted food groups [34].

This paper presents the findings of the impact and process evaluation of the FEAST program. The primary aim of this trial was to assess the effectiveness of the program on F&V consumption among 10-12-year-old children. Secondary aims assessed F&V variety and the following food literacy constructs: nutrition knowledge, food preparation and cooking skills, self-efficacy and behaviours (i.e. preparing food [F&Vs and salads], following recipes, and frequency of cooking dinner at home); food waste knowledge and behaviours (i.e. willingness to eat ‘imperfect’ F&Vs, and daily food lunch box waste behaviours); as well as food production knowledge (i.e. understanding the ‘farm to plate’ concept). It was hypothesised that students undertaking the FEAST program compared to students in the wait-list control (WLC) schools would increase F&V consumption, and improve food literacy knowledge, skills and behaviour. The process evaluation examined: the reach (to students); adoption (by schools); implementation (training of teachers, adherence to program implementation; barriers and facilitators); maintenance; satisfaction; and perceived benefits (by teachers and students).

## Methods

### Study design and participants

The TREND statement [35], in conjunction with the CONSORT 2010 [36], were used as a guide to report this evaluation study (See Additional file A). This study was approved by the Human Ethics Advisory Group, Faculty of Health at Deakin University (HEAG-H-31_2020), as well the Department of Education, via the NSW State Education Research Application Process (SERAP 2019163 ). This trial was prospectively registered with the Australian and New Zealand Clinical Trials Registry (ACTRN12620001347954) on the 14th of December 2020. All methods were carried out in accordance with relevant guidelines and regulations of *The Declaration of Helsinki*. Full details of the methodology have been previously published [37].

Briefly, this was a cluster non-randomized controlled trial (NRCT) in which 177 primary schools were invited to participate in the program’s evaluation, following their registration with OzHarvest, to implement the FEAST program in their schools. Twenty schools enrolled in this study, 10 schools agreeing to participate as intervention schools and 10 as WLC schools (delayed intervention). The intervention was implemented partially face-to-face in the classroom setting and partially online in the home setting, due to COVID-19 school closures, in the 2021 Australian scholastic year. This was a deviation from the original design as outlined in the published protocol [37]. All schools met the following inclusion criteria: (i) participating in the FEAST program in 2021; (ii) students were in Grades 5–6 or were between 10 and 12 years of age; and (iii) students had a school email address. Schools were excluded if: (i) they had previously participated in the FEAST program or (ii) were schools that catered exclusively to children with special needs.

All students and parents at the recruited schools were contacted to participate via information sent home by the classroom teachers. Although the FEAST program was curriculum-integrated and did not require consent, parents/carers could provide written informed opt-out consent if they did not want their child to participate in the evaluation (i.e. if they did not want their child to complete the two FEAST surveys). Students were also advised that they could opt out during data collection.

### Sample size

Details of the sample size calculations have been published previously [37]. Briefly, calculations indicated recruitment of 20 schools (10 per intervention arm), with an average of 50 students (SD ± 22) per school would provide 97% and 90% power (α = 0.025) to detect a 0.5 serves/day group difference in fruit and vegetable intakes (respectively). For the primary outcomes, an increase of 0.5 servings of fruit/day (i.e. 75 g) and vegetables/day (i.e. 37.5 g) was considered to be meaningful [38, 39].

### Intervention

FEAST was a primary-school, classroom-based, curriculum-aligned program that used inquiry-based approaches to learning, which were interactive and student-centred [40]. FEAST was integrated with lessons mapped to the Australian Curriculum, embracing Grade 5–6 key learning areas (KLAs) and the cross-curricular priority of sustainability [41]. The PRECEDE-PROCEED Planning model (PPM) [42] and Social Cognitive Theory (SCT) [43] were used to develop FEAST. This program was based on the following SCT components: behavioural capability, outcome expectations, self-efficacy, observational learning and role modelling [43].

Classroom teachers undertook face-to-face or online training with OzHarvest before implementing the FEAST program. OzHarvest provided resources and support to teachers to deliver the 1.5-hour lesson/week for one school term (i.e. 10-weeks). The ten theory components included lessons on: healthy food (e.g. food groups, F&Vs); food waste (e.g. how to reduce food waste, fridge and fruit bowl audit; how OzHarvest rescues food, preventing it going to landfill); food production ‘farm to plate’ concepts (e.g. where food comes from); and designing recipes using commonly wasted foods such as F&Vs (e.g. utilizing bruised bananas to make banana pikelets/bread/muffins). The six practical components included: food safety; food preparation skills; using/designing recipes; collating a class cookbook; cooking hot/cold meals; and tasting foods prepared with classmates, families and/or volunteers. FEAST was consistent with the Australian Dietary Guidelines and state/territory-based healthy eating strategies [44], and all recipes included fruits and/or vegetables (e.g. fruit skewers with natural yoghurt, tzatziki yoghurt dip with vegetable sticks, fast (vegetable) fritters, rainbow (vegetable) honey soy noodle stir fry, crunchy noodle salad). Details of the program training modules, educational resources, lesson plans and practical guide components have been outlined in the previously published protocol [37].

The FEAST program was implemented during Term 3 (12 July-17 September) of the 2021 scholastic year. This coincided with COVID-19 public health measures in the state of New South Wales (NSW) that enforced stay at home orders with subsequent school closures and ensuing home schooling. Given the significant disruptions of lockdowns, school teachers and OzHarvest adapted the program to an online teaching platform. Consequently, FEAST was implemented both remotely (i.e. on-line) by the teachers, and in the classroom setting when schools resumed face-to-face teaching. Because of school closures in Term 3, FEAST was completed in Term 4. As a result, each intervention school had their own start and completion dates as well as baseline and post-intervention data collection, deviating from the original protocol [37].

### Wait-list control

Due to the challenges of recruiting schools during the pandemic, OzHarvest provided funding opportunities (covering costs of the FEAST online teacher training, educational resources and kitchen kits) from local government, corporate or philanthropic organisations to incentivise disadvantaged schools in regional NSW to participate as WLC schools. The WLC schools continued with their regular academic program. Baseline data collection dates occurred in Term 3, and post-intervention data collection occurred in Term 4. The WLC schools received the intervention during the 2022 scholastic year (no data were collected).

### Outcome measures

####  Student surveys

FEAST was evaluated using a 25-item self-reported survey, which included questions on nutrition/intake; food preparation/cooking and food waste/production. The primary outcomes included fruit and vegetable consumption (serves/day). Secondary outcomes included the proportion of children reporting eating the recommended (fruit) or above average (vegetable) intakes; nutrition knowledge, food preparation and cooking (skills, self-efficacy and behaviours); food waste (knowledge and behaviour); and food production (knowledge). A detailed description of all measures is provided in the published protocol [37]. A summary detailing items and survey development, using previously published reliable and validated measures (where possible), can be found in Additional file 1. The survey also included questions capturing demographic information such as age, gender, grade and language/s spoken at home. All data were captured via the REDcap platform [45].

### Teacher surveys

Surveys were only issued to teachers who implemented the FEAST program post-intervention to seek feedback and to aid in the process evaluation. The teachers’ survey also included questions capturing basic demographic information of the teachers, as well as questions related to: injuries sustained by students during FEAST cooking activities (i.e. harms assessment); extra-curricular activities undertaken by their classes (such as nutrition and sustainability-related programs), and school policies (such as healthy canteen and/or sustainability policies). Costs relating to the FEAST program have also been outlined.

Additional files 2 and 3 present the student and teacher FEAST evaluations (respectively) for intervention schools only, which incorporated additional questions to capture COVID-19-related issues.

### Parent/carer and volunteer surveys

Initially the process evaluation was designed to gain feedback from parents/carers and community volunteers. However, due to COVID-19 public health orders and multiple lockdowns in the state of NSW during 2021, it was decided that it would be best to engage only students and teachers, so as to minimise the additional burden on parents/carers during pandemic lockdowns. Also, when schools returned to face-to-face lessons neither parents/carers nor community volunteers were permitted to participate in the FEAST cooking activities in school, due to pandemic social distancing requirements.

### Process evaluation

Details of the process evaluation protocol have been previously published [37]. Briefly, the RE-AIM framework (Reach, Efficacy, Adoption, Implementation and Maintenance) [46] was utilized to guide the process evaluation with the inclusion of two additional parameters. The following parameters were assessed: reach (to students); adoption (by schools); implementation (training of teachers, adherence to program implementation by teachers; barriers and facilitators); maintenance (intention by students and teachers); satisfaction (by teachers and students); and perceived benefits (by teachers and students). These data were collected post-intervention via: (i) surveys issued to teachers who implemented the FEAST program; (ii) an additional section of questions appended to student surveys for those who completed the program; and (iii) administrative data from OzHarvest.

### Statistical analyses

Statistical analyses were undertaken using Stata 17.0 BE (Basic Edition) [47]. Descriptive statistics (mean ± SD or n [%]) were calculated for student and school level baseline characteristics according to intervention group. Group differences were assessed using t-tests (continuous variables) and chi-square tests (categorical variables).

To estimate intervention effects on primary and secondary outcome measures, random-intercept generalised linear mixed models (GLMM), which account for repeat observations nested within individuals, within schools, were fitted using appropriate family and link functions according to outcome type. Models included main effects of time (baseline/post-intervention) and intervention group and their interaction, with the interaction term used to estimate group differences for changes in outcomes from baseline to post-intervention. For example, for the continuous primary outcomes, this quantity represented the group difference in mean pre-post change of fruit/vegetable intakes. Intervention effects were reported as mean differences for continuous outcomes and odds ratios (ORs) for categorical outcomes. The GLMMs were adjusted for *a priori* determined [37] confounders including age, sex, students speaking another language at home, and school’s Index of Community Socio-Educational Advantage (ICSEA). The ICSEA values range from around 500 representing schools from extremely disadvantaged backgrounds to about 1300 representing schools with students from very advantaged backgrounds (mean of 1000 ±100) [48].

Two changes were made for the choice of confounders from the published protocol. Firstly, student grade was substituted for age. This occurred because in Australia, some regional schools with small student numbers combine several grades into one classroom. Additionally, some students are accelerated to higher grades based on academic achievements. The potential confounder teacher training (face-to-face vs. online) was not included in the model, as outlined in the published protocol, as this only applied to the intervention group (not the WLC group) and was inconsequential to the analysis.

Primary analyses of intervention effectiveness were conducted on an intention-to-treat basis and included all participants who provided baseline and/or follow-up data (18 schools; *n* = 977 students; 1391 observations). Longitudinal mixed models using all available repeated-measures data can provide valid estimates under a missing at random assumption of the missing data, and there is evidence that for studies with a high percentage of missing values, the mixed model approach without ad hoc imputations is more powerful than other options [49] and a more sophisticated approach to handle missing data [50].

The results of the trial have been reported using two types of analysis: available case analysis (ACA) and complete case analysis (CCA) using GLMMs. The primary analysis, the ACA used all available data points from participants from 18 schools (*n* = 977). The CCA was used to assess sensitivity of the findings to different assumptions around the missing data mechanism (valid under a missing completely at random assumption). As such, the CCA used matched data points from participants who had completed both pre and post surveys (16 schools, *n* = 432). As there were two primary outcomes, a Bonferroni-corrected alpha (α) of 0.025 was used to indicate statistical significance. For all other analyses, statistical significance was set at *p* < 0.05.

Analyses for the process evaluation, involving student and teacher responses, in addition to data provided by OzHarvest, have been presented as descriptive statistics for the quantitative component, using standard summary statistics.

### Blinding

Although it is not possible to blind participants in implementation studies [51], the statistician was blinded to group allocation.

## Results

**Fig. 1:**
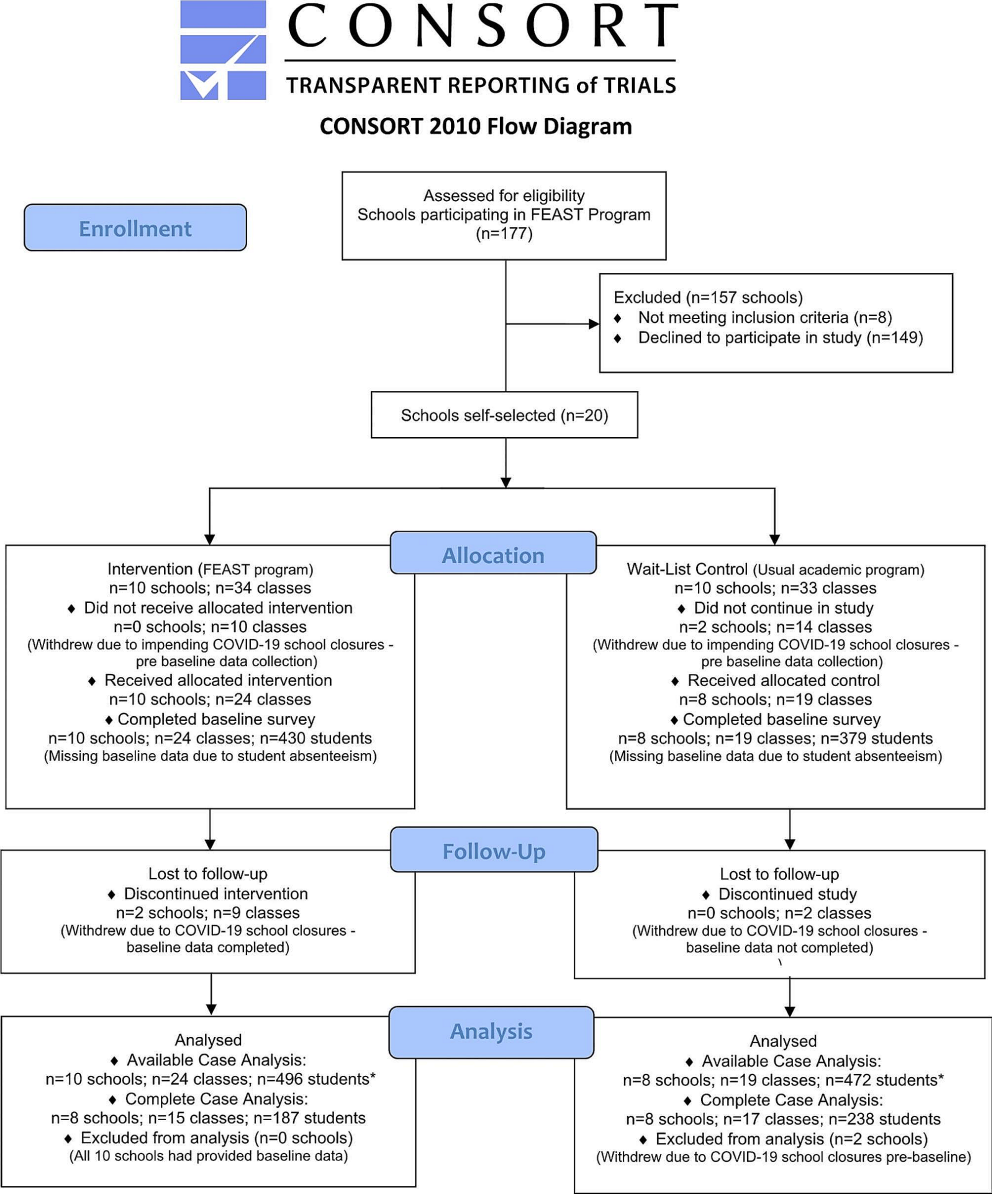
*Student numbers higher than baseline, as absent students provided post data only and were included in Available Case Analysis using Intention-to-Treat principles (numbers based on primary outcome data)

The CONSORT flow diagram [52] in Fig. 1, shows enrolment and participation in the FEAST program study. Of the 20 schools that agreed to participate in the FEAST evaluation, two WLC schools withdrew before baseline data collection citing challenges due to COVID-19 school closures. Of the 978 eligible children at the 18 remaining schools, 977 children (or 99.9%) were available to participate in the study. Informed opt-out consent was obtained from all the children’s parents/carers. Only one parent/guardian withdrew consent via the opt-out process. In total, 809 students (i.e. *n* = 430 intervention group [53.2%]; *n* = 379 WLC group [46.8%]) completed baseline data (82.8%). At study completion, 600 students (74.2% of baseline participants) completed post-intervention surveys (intervention group *n* = 260, WLC group *n* = 340).

Descriptive statistics of baseline student and school demographics and primary outcomes of interest for the intervention and WLC schools are presented in Table 1. Of the seven demographic variables reported, there were significant differences between intervention and WLC schools for the student variables (age, grade, other language spoken at home) and school variables (school type and geographical location). Intervention students on average were older and more likely to be in Grade 6, only speak English at home, attend a non-government school, and live in a major city. There were no baseline differences in student sex or ICSEA. The primary outcomes of interest also showed no differences at baseline (for Intervention vs. WLC group: fruit serves/day − 2.25 (± 1.01) vs. 2.23 (± 0.92) and for vegetable serves/day − 2.39 (± 1.33) vs. 2.50 (± 1.35)).

**Table 1:** Baseline student and school characteristics and primary outcomes between intervention and wait-list control groups

	Total Sample *N* = 809 18 Schools	Intervention Grp *N* = 430 10 Schools	Wait-List Control Grp *N* = 379 8 Schools	*p*-value*
Student Characteristics				
Age:				
Years (Mean ± SD) Age range	11.07 (0.71) 9–13 years	11.10 (0.07) 9–13 years	10.91 (0.70) 10–13 years	< 0.001*
Sex: n (%)				
Male Female Prefer not to say	376 (46.5) 404 (49.9) 29 (3.6)	204 (47.4) 210 (48.8) 16 (3.7)	172 (45.4) 194 (51.2) 13 (3.4)	0.797
Grade: n (%)				
Year 6 Year 5 Year 4	519 (62.9) 286 (35.4) 14 (1.7)	311 (72.3) 106 (24.7) 13 (3.0)	198 (52.2) 180 (47.5) 1 (0.3)	< 0.001*
Other language spoken at home:				
Yes n (%)	188 (23.2)	116 (27.0)	72 (19.0)	0.008*
School Characteristics*				
Type of School: *n* of school type [*n* (%) of students]				
Government Non-Government	13 [572 (70.7%)] 5 [237 (29.3%)]	6 [234 (54.4%)] 4 [196 (45.5%)]	7 [338 (89.2%)] 1 [41 (10.8%)]	< 0.001*
Geographic Location: *n* of schools in that location [*n* (%) students]				
Major City Regional	9 [390 (48.2%)] 9 [419 (51.8%)]	6 [263 (61.2%)] 4 [167 (38.8%)]	3 [127 (33.5%)] 5 [252 (66.5%)]	< 0.001*
ICSEA Score: *n* (%) of schools in range				
700–800	1 (5.6%)	0 (0.0%)	1 (12.5%)	
801–900	2 (11.1%)	2 (20.0%)	0 (0.0%)	0.096
901–1000 1001–1100 1101–1200 Range of scores	10 (55.6%) 2 (11.1%) 3 (16.7%) 771–1171	5 (50.0%) 0 (0.0%) 3 (30.0%) 854–1171	5 (62.5%) 2 (25.0%) 0 (0.0%) 771–1090	
Primary Outcomes				
Fruit Intake: Mean (± SD) serves/day	2.24 (0.99)	2.25 (1.01)	2.23 (0.92)	0.642
Vegetable Intake: Mean (± SD) serves/day	2.44 (0.99)	2.39 (1.33)	2.50 (1.35)	0.267

**Legend**: *GRP* Group; School Characteristics* were accessed from the the *‘My School’* website - Australian Curriculum, Assessment and Reporting Authority (ACARA), Australian Department of Education website^48^; *ICSEA* Index of Community Socio-Educational Advantage, higher scores representing schools with students from more advantaged backgrounds (mean 1000 ± 100)) ^48^; *p* values were calculated using t-tests for continuous variables and chi-square tests for categorical variables; *p value set at 0.05 significance level for student and school characteristics

### Primary outcomes

Table 2 shows the estimated intervention effects of FEAST (Intervention group) compared to WLC group on primary outcomes (F&V intakes). Post-intervention, the Intervention group compared to WLC group consumed 2.24 vs. 2.33 serves of fruit/day, and 2.48 vs. 2.52 serves of vegetables/day (respectively). According to the ACA, there were no statistically significant intervention effects on F&V intakes. The sensitivity analysis supported these findings. However, both groups maintained F&V consumption at, or slightly above the national averages (i.e. 2.2 serves of fruit/day and 2.0 serves of vegetables/day) [53].

**Table 2:** Effects of FEAST compared to wait-list-control on primary outcomes (F&V intakes)

	Intervention (FEAST Program) 10 Schools		Wait-List Control 8 Schools		Available Case Analysis^a,b^ 18 Schools			Complete Case Analysis^a,c^ 16 Schools		
	*N* = 430	*N* = 260	*N* = 379	*N* = 340		*N* = 968			*N* = 425	
Primary Outcomes	Pre Mean ± SD	Post Mean ± SD	Pre Mean ± SD	Post Mean ± SD	Effect Size	95% CI	*p* value*	Effect Size	95% CI	*p* value*
Fruit	2.25 (1.01)	2.24 (0.92)	2.23 (0.97)	2.33 (0.92)	-0.04	-0.20, 0.12	0.642	-0.03	-0.20, 0.14	0.750
Vegetable	2.39 (1.33)	2.48 (1.28)	2.50 (1.35)	2.52 (1.39)	0.13	-0.10, 0.35	0.267	0.14	-0.11, 0.38	0.287

**Legend**: ^a^Generalised Linear Mixed Models (*GLMM*) used to assess intervention effects; GLMMs, adjusted for sex, age, language, ICSEA (school’s Index of Community Socio-Educational Advantage) presented as mean adjusted change (*95% CI* 95% Confidence Intervals) in primary outcomes for children exposed to intervention relative to control; ^b^ Available Case Analysis (using Intention-to-Treat principles) and ^c^ Complete Case Analysis (using matched pre and post data available for variables analyzed); *SD* Standard Deviation; ^*^p value set at 0.025 (Bonferroni-corrected); *Fruit* Intakes Range 0–4 serves/day; *Vegetable* Intakes Range 0–5 serves/day

### Secondary outcomes

Tables 3 and 4 show the estimated intervention effects of the FEAST program compared to WLC on the continuous and categorical secondary outcomes, respectively. Results showed no significant between-group differences in any of these outcomes. The sensitivity analysis supported these findings except for one outcome. There was a statistically significant intervention effect for the ‘farm to plate’ outcome: the proportion of Intervention group participants understanding the ‘farm-to-plate’ concept increased from pre- to post-intervention, relative to the WLC group (OR 4.11 [95% CI 1.07, 15.84] *p* = 0.040).

**Table 3:** Effects of FEAST compared to wait-list-control on secondary continuous outcomes (behaviour and self-efficacy)

	Intervention (FEAST Program) 10 Schools		Wait-List Control 8 Schools		Available Case Analysis^a,b^ 18 Schools			Complete Case Analysis^a,c^ 16 Schools		
	*N* = 430	*N* = 260	*N* = 379	*N* = 340		*N* = 977			*N* = 425	
Secondary Outcomes: Continuous	Pre Mean (± SD)	Post Mean (± SD)	Pre Mean (± SD)	Post Mean (± SD)	Effect Size	95% CI	*p* value*	Effect Size	95% CI	*p* value*
NUTRITION F&V Variety Consumed: Behaviour										
Fruit variety	2.77 (2.39)	3.08 (2.46)	2.72 (2.07)	3.25 (2.67)	-0.09	-0.54, 0.36	0.692	0.14	-0.36, 0.63	0.590
Vegetable variety	4.02 (3.83)	4.03 (3.49)	3.85 (3.31)	4.51 (4.16)	-0.17	-0.76, 0.41	0.568	0.04	-0.58, 0.66	0.901
COOKING SKILLS: Self-efficacy										
Cooking Skills Score	5.83 (1.59)	6.15 (1.27)	5.75 (1.46)	5.98 (1.42)	0.08	-0.13, 0.28	0.460	0.02	-0.20, 0.24	0.859
FOOD WASTE: Behaviour										
School Lunch Eaten	3.31 (0.84)	3.29 (0.85)	3.19 (0.90)	3.17 (0.95)	-0.04	-0.19, 0.12	0.637	-0.10	-0.27, 0.07	0.270
Banana Choice Score	2.17 (1.56)	2.62 (1.72)	2.11 (1.47)	2.37 (1.56)	0.22	-0.07, 0.50	0.134	0.19	-0.13, 0.52	0.248

**Legend**: ^a^Generalised Linear Mixed Models (*GLMM*) used to assess intervention effects; GLMMs, adjusted for sex, age, language, ICSEA (school’s Index of Community Socio-Educational Advantage) presented as mean adjusted change (*95% CI* 95% Confidence Intervals) in secondary outcomes for children exposed to intervention relative to control; ^b^ Available Case Analysis (using Intention-to-Treat principles) and ^c^ Complete Case Analysis (using matched pre and post data available for variables analyzed); *SD* Standard Deviation; ^*^p value set at 0.05; Fruit variety consumed yesterday Mean (± SD) Score Range 0–14 [choice of 14 different fruits]; Vegetable variety consumed yesterday Mean (± SD) Score Range 0–24 [choice of 24 different vegetables]; Cooking Skills Mean (± SD) Sum of skills (I can make fruit/vegetable snack; salad; cut food; measure ingredients; follow recipe; help with family meal) Score Range 0–7; School Lunch Eaten (A little = 1, Half of it = 2 Most of it = 3, All of it = 4) Score Range 1–4; Banana Score - Willingness to eat or use in a recipe, perfect and imperfect bananas, Range 0–7 (unripe green bananas, perfect yellow bananas, blemished and bruised bananas)

**Table 4:** Effects of FEAST compared to wait-list-control on secondary categorical outcomes (behaviour and knowledge)

	Intervention (FEAST Program) 10 Schools		Wait-List Control 8 Schools		Available Case Analysis^a,b^ 18 Schools			Complete Case Analysis^a,c^ 16 Schools		
	*N* = 430	*N* = 260	*N* = 379	*N* = 340		*N* = 977			*N* = 425	
Secondary Outcomes: Categorical	Pre	Post	Pre	Post	Odds Ratio	95% CI	*p* value*	Odds Ratio	95% CI	*p* value*
NUTRITION: F&Vs Consumed										
N (%) consuming ≥ 2 fruit serves/day	332 (78.1)	215 (83.3)	291 (7.6)	275 (82.1)	1.23	0.50, 2.98	0.652	1.27	0.48, 3.37	0.628
N (%) consuming ≥ 2 vegetable serves/day	301 (71.3)	200 (77.5)	279 (74.2)	252 (75.2)	1.72	0.85, 3.47	0.133	1.75	0.76, 3.99	0.186
NUTRITION: Knowledge										
N (%) knowing recommendation of 2 serves of fruit/day	152 (36.5)	100 (39.4)	131 (35.1)	114 (34.2)	1.02	0.55, 1.88	0.958	§	§	§
N (%) knowing recommendation of 5 serves of vegetables/day	75 (17.7)	41 (15.9)	67 (17.8)	61 (18.1)	1.10	0.49, 2.50	0.817	1.52	0.58, 4.01	0.397
COOKING SKILLS: Behaviour										
N (%) help cook dinner at home	387 (92.1)	244 (94.9)	349 (93.6)	319 (94.9)	0.56	0.07, 4.74	0.596	0.32	0.03, 3.38	0.342
FOOD WASTE: Behaviour										
N (%) reporting eating imperfect F&Vs	260 (65.6)	176 (71.0)	197 (55.8)	210 (65.4)	0.88	0.47, 1.64	0.687	1.05	0.49, 2.27	0.894
FOOD WASTE: Knowledge										
N (%) knowing food waste impacts environment	237 (57.7)	163 (64.4)	193 (52.3)	182 (55.0)	1.31	0.72, 2.38	0.369	1.88	0.92, 3.85	0.083
FOOD PRODUCTION: Knowledge										
N (%) knowing ‘farm-to-plate’ concept	360 (89.1)	227 (90.8)	321 (88.2)	283 (86.8)	2.38	0.85, 6.69	0.099	4.11	1.07, 15.84	0.040*

**Legend**: ^a^Generalised Linear Mixed Models (*GLMM*) used to assess intervention effects, adjusted for sex, age, language, ICSEA (school’s Index of Community Socio-Educational Advantage) presented as mean adjusted change (*95% CI* 95% Confidence Intervals) in secondary outcomes for children exposed to intervention relative to control; ^b^Available Case Analysis (using Intention-to-Treat principles) and ^c^Complete Case Analysis (using matched pre and post data available for variables analyzed); *SD* Standard Deviation; ^*^p value set at 0.05; *NUTRITION F&V Consumed* Number and percentage N (%) of children consuming ≥ 2 fruit serves/day and ≥ 2 vegetable serves/day; *NUTRITION: Knowledge* Number and percentage N (%) of children knowing recommendation of 2 serves of fruit/day and 5 serves of vegetables/day; *COOKING SKILLS: Behaviour* Number and percentage N (%) of children helping family cook dinner at home (i.e. once in a while to almost every night); *FOOD WASTE: Behaviour* Number and percentage N (%) of children reporting eating imperfect F&Vs; *FOOD WASTE: Knowledge* knowing food waste impacts environment; *FOOD PRODUCTION*: Knowledge Number and percentage N (%) of children knowing ‘farm-to-plate’ concept (i.e. knowing a strawberry travels from farm, on transport, to supermarket, to consumer’s plate); § this outcome had insufficient variability and thus no inferential analyses could be conducted as convergence could not be achieved for the CCA

### Process evaluation

From the 10 intervention schools that participated in the FEAST program evaluation, nine teachers from eight schools completed surveys. Two teachers withdrew their classes from the evaluation after the initial baseline data collection and program commencement, citing COVID-19 school closure challenges. These two teachers, however, did inform the primary investigator and OzHarvest’s FEAST team of the number of theory lessons undertaken before they withdrew their school from the evaluation. Table 5 describes the demographic profile of schools, classes and students participating in the process evaluation. Student and teacher survey results are outlined in Additional files 4, 5, 6, 7, 8, 9, 10 and 11.

**Table 5:** FEAST intervention school teacher surveys - school, class, teacher, student and program data

School ID	Teacher Gender	Teacher training method	Class Nos enrolled pre-school closures	School Term FEAST conducted in	Grade FEAST implemented in	% of students participated in FEAST	No. of surveys each teacher completed	Baseline Class Nos.	Post FEAST Class Nos.	Baseline Student Survey Nos.	Post FEAST Student Survey Nos.	No. of Theory Lessons: Total	No. of Cooking Activities: Total	No. of Cooking Activities in class	No. of Cooking Activities at home
Teacher Survey Information															
1	F	ONL	4	T3-4	6	74.1	1	3	2	69	46	9	6	0	6
3	F	ONL	4	T3-4	4|5	100.0	4	4	4	70	59	3	0	0	0
3					5|6 a	100.0						3	0	0	0
3					5|6 b	88.0						3	0	0	0
3					5|6 c	100.0						3	0	0	0
4	F	ONL	4	T3-4	5|6	95.5	1	3	2	50	33	6	8	6	2
7	F	F2F	3	T3-4	5|6 a	100.0	3	3	1	40	20	5	0	0	0
7					5|6 b	100.0						6	2	0	2
7					5|6 c	100.0						6	1	0	1
8	F	F2F	1	T3-4	5	100.0	1	1	1	32	30	6	11	6	5
8	M		1	T3-4	6	100.0	1	1	1			6	11	6	5
12	F	ONL	1	T3-4	4,5,6	83.3	1	1	1	7	15	0	10	10	0
13		ONL	3	T3-4	4	100.0	1	1	1	8	7	0	3	3	0
18	F	ONL	6	T4	6a	100.0	2	2	2	42	50	8	7	4	3
18			4		6b	100.0						8	7	4	3
Communication with Primary Investigator and/or OzHarvest FEAST Education Team															
10	F	ONL	3	T3-4	6	NA	0	3	0	72	0	3	3	3	0
10											0	3	3	3	0
10											0	3	3	3	0
11	F	ONL	4	T3-4	5|6	NA	0	2	0	40	0	6	0	0	0
11											0	6	0	0	0
	F:M 10:1	ONL: (8 Sch) F2F: (2 Sch)	*N* = 34 classes originally enrolled	T3-4: (9 Sch) T4: (1 Sch)	Range Grades 4–6	Mean 95.9%	15 surveys 8 teachers	*N* = 24 classes provided baseline data	*N* = 15 classes provided post-data	*N* = 430 Students provided baseline data	*N* = 260 Students provided post-data	Theory Mean 4.6. SD ± 2.6	Cooking Total Mean 4.1 SD ± 4.3	Cooking in Class Mean 2.4 SD ± 3.2	Cooking at home Mean 1.5 SD ± 2.1

Legend: % Percentage, No. Number; Nos. Numbers; F Female; M Male; ONL Online; F2F face-to-face; T Term; 4|5 and 5|6 Composite class containing both grades; NA Not available; Sch School/s

### Reach

From the eight intervention schools, teachers reported that 356/371 students participated in the FEAST program, resulting in a reach of 95.9% (Table 5). Data on class size was not provided by the teachers that withdrew.

### Adoption (by schools)

Since 2018, 1752 primary schools in Australia have been contacted by OzHarvest and 643 have adopted the FEAST program (i.e. 36.7%).

### Implementation

#### Training of teachers

All teachers completed the FEAST training modules. Of the teachers that completed the post-FEAST teacher survey, three attended face-to-face training at OzHarvest and six completed online training.

#### Adherence to program implementation by teachers

Teachers implemented on average, 4.6 (SD ± 2.6) FEAST theory lessons ranging between 0 and 9 theory lessons (maximum 10, as per original protocol) (Table 5 and Additional file 4). Theory lessons were implemented by classroom teachers in the classroom setting (face-to-face, as per original protocol), and in the home setting (online, due to COVID-19 school closures). Teachers also implemented, on average 4.1 (SD ± 4.3) FEAST practical lessons (i.e. food preparation/cooking activities) ranging between 0 and 11 (although the original protocol stipulated six food preparation/cooking activities). The average number of food preparation/cooking activities implemented in the classroom setting was 2.4 (SD ± 3.2) (range 0–10) and in the home setting it was 1.5 (SD ± 2.1) (range 0–5) (Table 5).

#### Barriers to implementation

COVID-19 prohibited teachers from delivering the FEAST program as intended. They were unable to invite parents/carers or community volunteers to assist in helping and supervising food preparation/cooking activities, and restriction of movement in the classroom during social distancing rules made cooking sessions more challenging. One teacher cited ‘costs’ as a barrier. Some teachers reported students not having electronic devices at home and accordingly were not able to participate in online learning. For students who did not cook at home, barriers cited by teachers included: lack of access to ingredients, no parental/carer supervision and/or student disengagement.

#### Facilitators to implementation

Of the eight teachers who completed teacher survey questions relating to FEAST, seven agreed/strongly agreed that students found FEAST activities engaging, easy to follow, and resources easy to use in the classroom (Additional file 5). Six teachers found the program easy to integrate face-to-face into their daily classroom routine. All eight teachers agreed/strongly agreed that FEAST met their student’s learning needs, aligned with Grade 5–6 KLAs and the cross-curriculum priority of sustainability (Additional file 6). The three teachers who completed face-to-face training, and five of six teachers who completed online training agreed/strongly agreed it was effective and prepared them to deliver FEAST in the classroom setting (Additional file 7).

### Maintenance

As the evaluation process took place immediately post-intervention, it was not possible to measure the program’s long-term maintenance. The intention to maintain the program beyond initial implementation was assessed by asking the teachers “*will you continue implementing the FEAST program…?*” Eight teachers responded to this question, six indicating they would continue implementing FEAST, and two indicating they would not be able to due to moving out of their current roles. Of the 261 students, 192 (73.6%) indicated they would like to do FEAST again (Additional file 8).

### Satisfaction

Seven teachers responded to the question *“How likely are you to recommend FEAST?”* with five indicating *‘10, Extremely likely to Recommend’*, (mean 9.09 ± 1.57). Students answering the same question, produced a mean score of 7.0 (SD ± 4.6). For details on student satisfaction refer to Additional file 8. When teachers were asked what was their *“… favourite aspect of FEAST?”* eight teachers indicated they *‘loved’* cooking with their students and how much their students *‘loved’* and ‘*enjoyed’* the program; in particular cooking and eating the food prepared with their peers. The teachers noted students were surprised how much food was wasted, and that healthy food was also *‘tasty food’* (Additional files 7, 9, 10).

### Perceived benefits

Teachers cited students’ positive engagement i.e. they enjoyed seeing the *“growth… in students”; “development of… skills”; “change in their palette”; “new love… for cooking”; “appreciation of food waste”; “independent life skills”; “being able to cook cheap healthy meals for themselves”; “impact… on healthy habits for… students”;* and overall a *“great program, creating a love for food”* as the reason they would continue implementing the FEAST program beyond the study evaluation (Additional file 5). Additional file 11 provides details about the students (*n* = 172) who reported that they learnt new skills during the cooking activities.

### Harms Assessment

All teachers undertaking FEAST completed the program risk assessment prior to delivering the program to students. Responses indicated five students from two different schools were harmed during classroom food preparation/cooking activities. The injuries sustained were minor: cut while using a kitchen knife; injured while using a grater; and/or burnt while using an electric frying pan. No child suffered an allergic reaction during the tasting of the food prepared and cooked by the students.

### Cost

Fixed and variable costs were calculated for a class of 30 + students. Fixed costs included FEAST teacher training online ($100 AUD, which included the classroom curriculum package), kitchen kit ($645 AUD) and six electric frypans ($390 AUD), with the option to purchase OzHarvest aprons ($360 AUD). Variable costs (depending on class sizes and seasonal cost of ingredients) were $300 AUD, i.e. cost of ingredients to make six different recipes, according to OzHarvest’s estimates.

## Discussion

The FEAST intervention, compared to the WLC group, did not produce significant increases in outcomes of interest. Contrary to the hypothesis, there were no significant between-group differences in changes, in either the primary outcomes (F&V consumption) nor the secondary outcomes (F&V variety intakes, nutrition knowledge, food preparation and cooking skills, self-efficacy and behaviours, food waste knowledge and behaviours or food production knowledge). FEAST intervention participants were significantly more likely to understand the ‘farm-to-plate’ concept compared to WLC participants, but this finding was unsupported in the primary sensitivity analysis. Furthermore, the question for the ‘farm-to-plate’ concept was not taken from reliable and validated outcome measures. The process evaluation revealed that while the program was well liked by students and teachers, COVID-19 school closures prevented the program being delivered as per protocol [37].

Existing evidence on the effects of interventions like FEAST is mixed. Several studies conducted within the primary/elementary school setting have found that multi-component interventions including nutrition education and cooking activities, in addition to environmental sustainability education/activities (such as gardening, composting and procuring locally sourced produce) resulted in significantly increased fruit and/or vegetable consumption [54–61]. However, many other studies of this nature, like FEAST, found no significant increases in F&V consumption [27, 62–70].

There are several plausible explanations for the lack of significant intervention effects reported here. School-based studies that included cooking activities have reported positive impacts on children’s F&V consumption [71–74] and willingness to taste novel foods [75]. Several systematic reviews evaluating the impact of culinary interventions on dietary intake [76] and cooking activities in the primary school setting [77, 78], concluded that experiential activities involving preparation, cooking and tasting, improved healthy dietary behaviour [76–78], attitudes [76–78], self-efficacy [76] and knowledge [77]. Students participating in FEAST did not experience the program as per original protocol (due to COVID-19 pandemic school closures), resulting in limited exposure to food preparation, cooking activities, and recipe tasting in the classroom setting. This may explain the lack of significant differences between FEAST and WLC schools for cooking skills, self-efficacy, knowledge or behaviour change.

A systematic review investigating teaching approaches and strategies that promoted healthy eating in primary-school children reported improved F&V consumption in curriculum-based approaches that were used in addition to experiential learning activities and parental/carer involvement [19]. Furthermore, studies have demonstrated that interventions grounded in SCT have produced significant improvements in nutrition behaviours [79], such as increased F&V intakes [80, 81]. Although FEAST was curriculum-integrated and designed around SCT [43, 82], the observational learning and role modelling concepts that were planned to be incorporated into the classroom food preparation/cooking activities, did not eventuate (due to COVID-19 pandemic school closures). These activities were originally designed to be facilitated by teachers with the assistance of parents/carers and/or community volunteers. Even when schools in NSW were permitted to resume face-to-face teaching, non-school-staff adults were not permitted to enter school premises to assist in school activities due to social distancing and safety requirements. Accordingly, students participating in FEAST missed out on these key learning opportunities of observational learning and role modelling with their parents/carers and/or community volunteers. Additionally, parents/carers missed an opportunity to learn about nutrition and sustainable cooking with their children.

Process evaluations of other published multi-component interventions with non-significant results in F&V consumption also revealed that intervention schools did not implement programs as intended [62, 66, 67]. Most schools in those studies omitted several theory and/or hands-on experiential components, implementing less robust interventions [62, 66, 67]. This was consistent with our findings. This study’s process evaluation revealed that FEAST was not delivered as intended. Although the program was designed to be delivered as 10 modules for 1.5 h/week, including ten theory and six food preparation/cooking activities over one school term (i.e. 10 weeks), it was delivered sporadically over two school terms during COVID-19 pandemic school closures.

Teachers reported delivering on average 4.6 (SD ± 2.6) theory lessons and 4.1 (SD ± 4.3) food preparation/cooking activities. Some were delivered via online sessions involving teachers demonstrating cooking, and students learning with the assistance of their parents/carers at home. Teachers reported not all students participated during these online sessions, and for those who did participate, not all were able to attend all sessions. Teachers cited *‘student disengagement’*, ‘*lack of parental/carer supervision’* and *‘lack of access to home computers’* as reasons behind poor online attendance in some schools. One study investigating the psychosocial impacts of home-schooling during the pandemic reported that poor availability of resources (e.g. electronic devices and internet services) for schools and families had a negative impact on remote learning [83]. This was consistent with some of the teacher reports from the FEAST evaluation. While all teachers who participated during school closures perceived the program positively, all reported negative impacts on implementation, which was the major external factor impeding their students from undertaking the program fully.

Overall, the pandemic caused dramatic changes in the family home environment [84, 85], such that parents found themselves working from home as well as becoming responsible for their children’s distance education [85]. School closures were very disruptive to the lives of children and their parents/carers, causing high levels of psychological distress [83, 85, 86]. Mothers in particular had increased meal responsibilities during this time [85]. It has been well established that the home eating environment (e.g. family meals, food availability and parent feeding practices) impact children’s dietary intakes [87–89]. One study showed that children’s eating behaviours became less healthy during the pandemic, with decreased F&V intakes [90], while other studies found increases in high-calorie snack foods [86, 91–93]. A systematic review investigating the eating habits of children and adolescents during the pandemic confirmed that F&V consumption decreased, and unhealthy snacking and sweet consumption increased, despite increases in home-cooked meals and decreases in fast food consumption [84]. In one qualitative study, mothers reported that they had less rules around mealtime during this period [85], which could explain children’s less healthy eating behaviours. These are plausible explanations why children who participated in the FEAST program did not increase their F&V consumption compared to the WLC group.

It must be noted that collectively the cohort of students that participated in this study did not decrease their F&V consumption between baseline and post program evaluation (but maintained intakes at the national averages) under pandemic conditions, which included school closures. This could be attributable to the type of schools that are drawn to programs such as FEAST i.e. schools that are interested in healthy food and sustainability-related activities [94] as revealed by their extra-curricular activities. Such activities included schools having kitchen gardens; composting; healthy canteen and/or sustainability policies in place, among others, as outlined in Additional file 12.

A study including five Australian schools that targeted food waste reduction found a reduction in avoidable food waste (FW) [95]. That intervention targeted both school and home environments and used mixed methods for data collection, including food waste audits (to objectively determine FW outcomes) [95]. The intervention consisted of educational, skills-based, and whole-of-school-events, and targeted behaviour change to assist students’ involvement at home in choosing and/or preparing food to take to school [95]. These strategies resulted in reductions in avoidable FW [95]. This is contrary to our findings, in which FW behaviours were not significantly different between groups. FEAST provided two lesson plans for teachers on food waste education, which were amongst the most popular resources used during implementation (Additional file 4). However, the lack of targeted behaviour change strategies and whole-of-school involvement in the FEAST program, could explain the non-significant FW outcomes.

### Limitations and strengths

As mentioned previously, the major limitation of this study was that it was conducted during the COVID-19 pandemic, which caused many disruptions [96], such as the ensuing lockdowns with school-closures that educational institutions were unprepared for [97]. As such, the FEAST program was not implemented as intended, which likely influenced outcomes negatively [98, 99].

Another limitation was that our measures may not have been sufficiently sensitive to detect small differences. However, measures with previously established psychometric properties were used for primary outcomes and where available for secondary outcomes (see Additional file 1).

While school closures were the major barrier to delivering the FEAST program, we cannot rule out that other aspects may have contributed to the non-significant results. It is possible that the program did not target the right mediating factors with sufficient strength to effect behaviour change. Evidence collated from several systematic reviews [19, 77, 100, 101] and one umbrella review [102] shows that school-based nutrition programs that include experiential activities such as food preparation and cooking [19, 77, 100, 101], involved parents/carers [77, 100, 101], and were curriculum-integrated [19, 102], were more likely to produce increases in F&V consumption. While the FEAST program incorporated all of these evidence-based strategies, the strategy of implementing programs for six months or longer [16, 21, 101], was not included in the design. It is feasible that OzHarvest may need to increase the duration of the FEAST program to at least six months [16, 21, 101] in order to intensify exposure [21, 24], which could positively affect the desired behaviour changes.

Another limitation was that the study was not a randomized controlled trial. Schools self-selected to participate in the program’s evaluation depending on the timing of implementation, as this was a real-world program. It has been suggested that evidence-based public health interventions may need to use research designs other than RCTs, as RCTs are not always practical for evaluating these types of interventions [103]. The implementation of the FEAST program by OzHarvest provided an opportunity to evaluate a program that was incorporating both nutrition and sustainability concepts, in the primary school setting. As such, a pragmatic NRCT to gather data was designed around this implementation.

Strengths of the study include the controlled design and the mixed methods approach with qualitative questions embedded within the quantitative survey. Additionally, a process evaluation was conducted, allowing better understanding and interpretation of results of this impact evaluation [104]. The GLMM method, used to analyse outcomes of interest, is considered to be a powerful approach [49] to handle missing data [50]. The sample size estimations required approximately 1000 student participants and the final sample for the primary analysis (ACA) was 977 participants, hence the power was not impacted. Evidence regarding cost-effectiveness and return on investment of school-based health promotion programs is scarce [105]. However, it was beyond the scope of this study to produce an economic evaluation of the FEAST program, although costs of implementation were outlined. Finally, to our knowledge, this is the first study of this type to undertake a harms assessment.

Notwithstanding the results from this study, OzHarvest continues to implement the program to growing popularity. Since the first pilot study in 2018, FEAST has now been implemented in 836 primary (n=643) and secondary (n=193) schools across Australia. The program has also been recognised by the United Nations Global Compact Network in Australia as being capable of contributing to seven sustainable development goals for 2030 [106, 107]. Given the program was well-received and liked by students and teachers and its continued popularity, it warrants further investigation under more ideal circumstances. Investigating programs like FEAST, implemented to plan, have the potential to increase F&V intakes and improve food literacy knowledge, skills and behaviours among children, in addition to supporting the Australian Curriculum with health-promoting and sustainability messages and contribute to: health promotion within schools initiatives [108]; sustainable schools initiatives [109]; the national agenda to reduce food waste [110]; as well as contributing to the sustainable development goals for 2030 [107].

## Conclusion

This is the first large cluster non-randomized controlled trial designed to evaluate the FEAST program in the primary-school setting. This school-based curriculum-integrated nutrition and sustainability program with experiential cooking components was delivered during the COVID-19 pandemic and did not improve children’s F&V intakes or food literacy behaviours, knowledge or skills. However, the program was well-received and liked by students and teachers and warrants further investigation under less challenging implementation conditions. Improving children’s F&V intakes, albeit a challenging exercise, remains a priority worthy of further investigation.

### Electronic supplementary material

Below is the link to the electronic supplementary material.

Additional file 1: Survey items and development of survey for the FEAST Evaluation

Additional file 2: FEAST Evaluation

Additional file 3: Implementation 1

Additional file 4: Teacher surveys - FEAST lessons plans used by teachers during Implementation (*n* = 9 teachers, *n* = 15 class groups)

Additional file 5: Teacher Survey? teacher’s perceptions of student’s learnings during the FEAST program (*n* = 9 teachers)

Additional file 6: Teacher Survey? teacher’s rating FEAST educational content and program delivery (*n* = 9 teachers)

Additional file 7: Teacher surveys? teacher’s satisfaction with FEAST teacher training courses

Additional file 8: FEAST Student Surveys (Intervention Schools) - Satisfaction of Program *N* = 261

Additional file 9: Teacher Survey? Teacher satisfaction of FEAST resources during COVID-19 school-closures *n* = 9

Additional file 10: Teacher Survey? Teacher satisfaction of FEAST Resources (*n* = 9 teachers)

Additional file 11: FEAST Student Survey (Intervention Schools)? Skills learnt during food preparation and cooking activities (*n* = 172)

Additional file 12: Teacher Survey? reports of other nutrition and/or sustainability programmes implemented in intervention schools (*n*10 Schools)

## Data Availability

The datasets created and analyzed during the present study have been securely stored with the Deakin University Research Data Store and are available from the corresponding author upon reasonable request, subject to ethical approval.

## Abbreviations

FEAST: Food Education and Sustainability Training
F&V: Fruit and Vegetable
WLC: Wait-list control
GLMM: Generalised linear mixed models
NCD: Non-communicable disease
NRCT: Non-randomized controlled trial
KLA: Key Learning Areas
PPM: PRECEDE-PROCEED Planning Model
SCT: Social Cognitive Theory
REDcap: Research electronic data capture
RE-AIM: Reach, Efficacy, Adoption, Implementation and Maintenance
ICSEA: Index of Community Socio-Educational Advantage
ACA: Available Case Analysis
CCA: Complete Case Analysis
SD: Standard Deviation
AUD: Australian Dollar
