# Supplementary material for: Impact and process evaluation of a primary-school Food Education and Sustainability Training (FEAST) program in 10-12-year-old children in Australia: pragmatic cluster non-randomized controlled trial
Source: BMC Public Health. 2024 Mar 1;24:657. doi: 10.1186/s12889-024-18079-8 (PMC10905805; doi:10.1186/s12889-024-18079-8)
Supplement: Supplementary file 1 — Additional file 1: Survey items and development of survey for the FEAST Evaluation [file 12889_2024_18079_MOESM1_ESM.pdf]

Additional file 1: Survey items and development of survey for the FEAST Evaluation

Questions	Questions Source (REF)	Psychometric Assessment	Age	Cohort (country)	Survey completed	Response	Category
PRIMARY OUTCOMES							
**F&V Consumption**							
Fruit consumed<br>*How many fruits do you usually eat each day?*	Many Rivers Diabetes Prevention Project[1]<br><br>NSW Healthy Eating and Active Living Strategy, 2013–2018 [2]	ACAORN,[3] Assessed Criterion/concurrent/ predictive validity and reliability	10-12 years	Aboriginal and Torres Strait Islander and non-Indigenous rural children. (Australia)	Self-reported	Λ: Frequency Scale A	Behaviour Change Intake Core foods Fruit frequency
Vegetables consumed<br>*How many vegetables do you usually eat each day?*	Many Rivers Diabetes Prevention Project[1]<br><br>NSW Healthy Eating and Active Living Strategy, 2013–2018 [2]	ACAORN,[3] Assessed Criterion/concurrent/ predictive validity and reliability	10-12 years	Aboriginal and Torres Strait Islander and non-Indigenous rural children. (Australia)	Self-reported	Ψ: Frequency Scale B	Behaviour Change Intake Core foods Vegetables frequency
SECONDARY OUTCOMES							
**F&V Variety consumed**							
Fruit Variety<br>*Please indicate if you ate this fruit yesterday, by ticking the box or boxes that apply to you.*<br>List of 14 fruits.	Eat Well Be Active Program Modified Child Nutrition Questionnaire (CNQ) [4]	ACAORN,[3] Assessed Criterion/concurrent/ predictive validity and reliability	10-13 years	Mix of public and private, and metropolitan and rural primary schools. (Australia)	Self-reported	Tick if consumed	Behaviour Change Intake Core foods Fruit variety
Vegetable Variety<br>*Please indicate if you ate this vegetable yesterday, by ticking the box or boxes that apply to you.*<br>List of 24 vegetables.	Eat Well Be Active Program Modified Child Nutrition Questionnaire (CNQ) [4]	ACAORN,[3] Assessed Criterion/concurrent/ predictive validity and reliability	10-13 years	Mix of public and private, and metropolitan and rural primary schools. (Australia)	Self-reported	Tick if consumed	Behaviour Change Intake Core foods Vegetables variety
**Nutrition Knowledge**							
Fruit Recommendations<br>*How many serves of fruit should you eat every day?*	Eat Well Be Active Program Modified Child Nutrition Questionnaire (CNQ) [4]	ACAORN,[3] Assessed Criterion/concurrent/ predictive validity and reliability	10-13 years	Mix of public and private, and metropolitan and rural primary schools.	Self-reported	Φ: Frequency Scale C	Knowledge Daily recommendations of fruit intake

				(Australia)			
Vegetable Recommendations<br>*How many serves of vegetables should you eat every day?*	Eat Well Be Active Program<br>Modified Child Nutrition Questionnaire (CNQ) [4]	ACAORN,[3] Assessed Criterion/concurrent/ predictive validity and reliability	10-13 years	Mix of public and private, and metropolitan and rural primary schools.<br>(Australia)	Self-reported	Δ:<br>Frequency Scale D	Knowledge<br>Daily recommendations of vegetable intake
**Cooking**							
Cooking skills<br>*I can make fruit snack; vegetable snack; salad; help with family meal; cut up food; measure ingredients; follow recipe.*	Cooking with Kids program [5]	Validity-tested for self-efficacy	9-11 years	Children in 4th - 5th grade<br>(US)	Self-reported	Tick<br>Yes/No/<br>Unsure	Cooking Skills<br>Self-efficacy
Cooking Frequency<br>*How often do you help cook dinner at home?*	Experiential Cooking and Nutrition Education Program.[6]	Original items developed for study. [6]<br>No validity and/or reliability testing	8-14 years	Children in low income communities.<br>3rd –8th grade<br>(US)	Self-reported	Ξ:<br>Frequency Scale E	Behaviour Change<br>Self-efficacy
**Food Waste**							
On a normal day, how much of your lunch do you eat at school?	Tackling Avoidable Food Waste in Western Australian Schools: Final Report 2016 [7]	No validity and/or reliability testing.<br>Published [8]<br>Modified multiple choice answers by adding extra options.	10-14 years	Children in upper primary to lower high school in Western Australia.<br>(Australia)	Self-reported	Multiple Choice, Yes/No/ Unsure, Short answers	Behaviour Change<br>Attitude
Do you eat blemished or imperfect fruits and vegetables?	Designed by OzHarvest FEAST education team.	No validity and/or reliability testing.	10-12 years	Pilot tested in NSW, Australian public schools.<br>Grade 5-6.	Self-reported	Yes/No	Behaviour Change
Does food that is thrown away affect the environment?	Designed by OzHarvest FEAST education team	No validity and/or reliability testing.	10-12 years	Pilot tested in NSW, Australian public schools.<br>Grade 5-6.	Self-reported	Yes/No	Knowledge
Which bananas would you eat or use in a recipe?	Designed by OzHarvest FEAST education team.	No validity and/or reliability testing.	10-12 years	Pilot tested in NSW, Australian public schools.<br>Grade 5-6.	Self-reported	Tick - from choice of 7 images of bananas (from green, to perfect yellow, to	Behaviour Change

						blemished and bruised)	
**Food Production**							
Farm-to-Plate concept<br>*What is the correct order a strawberry travelled to get to your plate?*	Designed by OzHarvest FEAST education team.	No validity and/or reliability testing.	10-12 years	Pilot tested in NSW, Australian public schools. Grade 5-6.	Self-reported	Multiple choice	Knowledge

Legend: ACAORN: Australian Child and Adolescent Obesity Research Network; F Fruit; V: Vegetable; Rec Recommended servings, daily Intake;
Λ Frequency scale A: I don't eat fruit, 1 serve or less per day, 2 serves per day, 3 serves per day, 4 or more serves per day.
Ψ Frequency scale B: I don't eat vegetables, 1 serve or less per day, 2 serves per day, 3 serves per day, 4 serves per day, 5 serves or more serves per day.
Φ Frequency Scale C: I don't know, 1 serve, 2 serves, 3 serves, 4 serves or more.
Δ Frequency Scale D: I don't know, 1 serve, 2 serves, 3 serves, 4 serves, 5 serves or more.
Ξ Frequency Scale E: Never, Once in a while, A few times a week, Every night or almost every night

Secondary Outcomes and Creating New Variables

For the secondary outcomes, in order to calculate the proportion of children consuming the recommended 2 serves of fruit/day,[9] the fruit intake variable was dichotomised to represent 0-1 serves/day vs 2 or more serves/day. Given that on average, Australian children consume only 2 serves of vegetables/day, instead of the recommended 5 serves/day,[9] the vegetable intake variable was dichotomised to represent 0-1 serves/day vs 2 or more serves/day, in order to calculate the proportion of children consuming the national average or more. For the variable, variety of F&Vs consumed yesterday, two scores (total number of different fruits and of different vegetables, consumed yesterday) were created for each participant. Regarding the seven questions on cooking self-efficacy (I can make fruit snack; vegetable snack; salad; help with family meal; cut up food; measure ingredients; follow recipe), a composite score was created for each participant, by adding the questions students responded 'yes' to. For the question that measured cooking behaviours (i.e. how often they help family cook dinner), the variable was dichotomised to represent 'never' helps vs sometimes helps family cook (i.e. once a week through to almost every night in the week). To measure food waste behaviours, students were presented with images of seven bananas ranging from unripened through to perfect yellow bananas through to bruised/blemished bananas. A score for each participant was created based on the total number of bananas they reported being willing to eat.

References:

1. Gwynn JD, Flood VM, D'Este CA, et al. The reliability and validity of a short FFQ among Australian Aboriginal and Torres Strait Islander and non-Indigenous rural children. Public Health Nutr. 2011;14(3):388-401.
2. Flood V GJ, Gifford J, Tuner N, Hardy L. Evidence on existing, validated short-form survey instruments for children’s diet, physical activity, and sedentary behaviour: an Evidence Check review brokered by the Sax Institute (wwwsaxinstituteorgau) for the NSW Ministry of Health. 2016.
3. Australian Child and Adolescent Obesity Research Network. ACAORN repository of tools to measure dietary intake in children and adolescents 2012 [Available from: http://anzos.com/assets/acaorn/Pub-Repository-diet-assess-tools-Sept2012.pdf.
4. Wilson AM, Magarey AM, Mastersson N. Reliability and relative validity of a child nutrition questionnaire to simultaneously assess dietary patterns associated with positive energy balance and food behaviours, attitudes, knowledge and environments associated with healthy eating. International Journal of Behavioral Nutrition and Physical Activity 2008;5:5.
5. Lohse B, Cunningham-Sabo L, Walters LM, et al. Valid and Reliable Measures of Cognitive Behaviors toward Fruits and Vegetables for Children Aged 9 to 11 Years. J Nutr Educ Behav. 2011;43(1):42-9.
6. Jarpe-Ratner E, Folkens S, Sharma S, et al. An Experiential Cooking and Nutrition Education Program Increases Cooking Self-Efficacy and Vegetable Consumption in Children in Grades 3–8. J Nutr Educ Behav. 2016;48(10):697-705.e1.
7. Boulet M, Wright B, M. R. Tackling Avoidable Food Waste in Western Australian Schools:Final Report 2016. BehaviourWorks Australia; Monash Sustainable Development Institute. Funded by the Waste Authority WA 2016.
8. Boulet M, Wright B, Williams C, et al. Return to sender: a behavioural approach to reducing food waste in schools. Australasian Journal of Environmental Management. 2019;26(4):328-46.
9. Australian Bureau of Statistics. National Health Survey: First Results, 2017-18 Canberra Commonwealth of Australia2018 [ABS Catalogue No. 4364.0.55.001]. Available from: https://www.abs.gov.au/ausstats/abs@.nsf/Lookup/by%20Subject/4364.0.55.001~2017-18~Main%20Features~Key%20Findings~1.
