# Supplementary material for: Impact and process evaluation of a primary-school Food Education and Sustainability Training (FEAST) program in 10-12-year-old children in Australia: pragmatic cluster non-randomized controlled trial
Source: BMC Public Health. 2024 Mar 1;24:657. doi: 10.1186/s12889-024-18079-8 (PMC10905805; doi:10.1186/s12889-024-18079-8)

FEAST Evaluation

What do you think about FEAST?

	Yes	No
Were the FEAST activities easy to read?	◯	◯
Were the FEAST activities easy to understand?	◯	◯
Was the FEAST website easy to use?	◯	◯
Was the FEAST program fun?	◯	◯

During school closures, when you were learning from home ...

	Yes	No
Were the FEAST lessons easy to do from home?	◯	◯
Did you do the FEAST lessons online?	◯	◯
Did you cook some of the FEAST recipes with your family at home?	◯	◯
Were the FEAST cooking activities easy to do at home?	◯	◯
Did you enjoy cooking with your family?	◯	◯
Would you like to continue cooking with your family?	◯	◯

Cooking with Classmates

Did you participate in the cooking activities at school with your class?

◯ Yes
◯ No

Did you enjoy cooking in class with your classmates?

◯ Yes
◯ No

Did you learn something about food preparation and cooking that you did not know before?

◯ Yes
◯ No

Name one new thing you learnt to do during the FEAST cooking activities.

Which was your favourite FEAST recipe?

Choose as many as you like.

- ☐ Peach Parfait
- ☐ Fruit Skewers with Natural Yoghurt
- ☐ Muesli Bliss Balls
- ☐ Tzatziki Dip with Vegetable Sticks
- ☐ Quick Pickle Vegetables
- ☐ Bircher Muesli with Apples and Bananas
- ☐ Rainbow Salad Roll
- ☐ Sandwich Sushi
- ☐ Chicken & Lentil Kofta Pita Pockets
- ☐ Crunchy Noodle Salad
- ☐ Banana Pikelets
- ☐ Turkish Carrot & Yoghurt Dip
- ☐ Fast Fritters
- ☐ From-the-Fridge Omelette
- ☐ French Toast
- ☐ Wholemeal Burrito Wrap
- ☐ Rainbow Honey Soy Noodle Stir Fry
- ☐ San Choy Bau
- ☐ Tortilla Wraps
- ☐ Butter Bean Hummus
- ☐ None of them

Did your class create a cookbook?

- ○ Yes
- ○ No

Did you enjoy creating the cookbook?

- ○ Yes
- ○ No

On a scale of 0 to 10, how likely are you to recommend the FEAST program to others?

- ○ 10, Extremely likely to Recommend
- ○ 9
- ○ 8
- ○ 7
- ○ 6
- ○ 5
- ○ 4
- ○ 3
- ○ 2
- ○ 1
- ○ 0, Not likely to Recommend

Would you like to do the FEAST program again?

◯ Yes
◯ No

Remember to click on the "FINISH" button

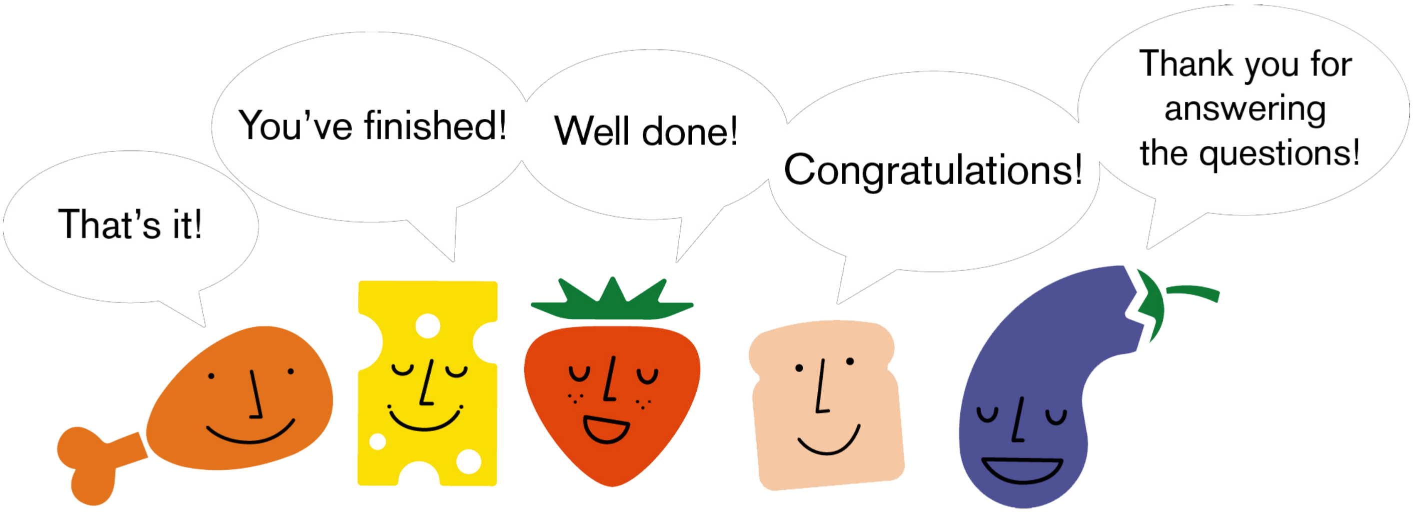

Supplement: Supplementary file 2 — Additional file 2: FEAST Evaluation [file 12889_2024_18079_MOESM2_ESM.pdf]
