# Supplementary material for: Impact and process evaluation of a primary-school Food Education and Sustainability Training (FEAST) program in 10-12-year-old children in Australia: pragmatic cluster non-randomized controlled trial
Source: BMC Public Health. 2024 Mar 1;24:657. doi: 10.1186/s12889-024-18079-8 (PMC10905805; doi:10.1186/s12889-024-18079-8)
Supplement: Supplementary file 3 — Additional file 3: Implementation 1 [file 12889_2024_18079_MOESM3_ESM.pdf]

Implementation 1

Implementation Details

Class [ft_classlist_01]
[ft_classno_01] Students
[ft_classppt_01] Students Participated

Did COVID19 school closures, impact the implementation of the FEAST Program in this class?

◯ Yes
◯ No. Please explain ______

Did you conduct any of the FEAST Program during COVID19 school closures?

◯ YES, all of it.
◯ YES, some of it.
◯ NO, the program stopped.

How did you provide the FEAST theory lessons to your students?

Please tick as many as apply.

☐ In the classroom setting
☐ In the student's home setting
☐ Combination (in both the classroom and the student's home)
☐ Other

Please specify what "Other" methods you have used to implement the program.

____

In the CLASSROOM setting, how many FEAST theory lessons did you conduct online?

◯ 0 ◯ 1 ◯ 2 ◯ 3 ◯ 4 ◯ 5 ◯ 6 ◯ 7 ◯ 8 ◯ 9 ◯ 10

In the CLASSROOM setting, how many FEAST theory lessons did you conduct using printed materials?

◯ 0 ◯ 1 ◯ 2 ◯ 3 ◯ 4 ◯ 5 ◯ 6 ◯ 7 ◯ 8 ◯ 9 ◯ 10

In the HOME setting, how many FEAST theory lessons did you conduct online?

◯ 0 ◯ 1 ◯ 2 ◯ 3 ◯ 4 ◯ 5 ◯ 6 ◯ 7 ◯ 8 ◯ 9 ◯ 10

For the HOME setting, how many FEAST theory lessons, did you print and send home to your students?

◯ 0 ◯ 1 ◯ 2 ◯ 3 ◯ 4 ◯ 5 ◯ 6 ◯ 7 ◯ 8 ◯ 9 ◯ 10

Which specific FEAST LESSON PLANS did you use?

Please tick as many as apply.

☐ Lesson 1: The essential question and scenario
☐ Lesson 2: Understanding food waste
☐ Lesson 3: Change-makers and where does food come from?
☐ Lesson 4: Using STEM Thinking for Problem Solving
☐ Lesson 5: Understanding food waste
☐ Lesson 6: Learning about healthy eating
☐ Lesson 7: Generating Ideas
☐ Lesson 8: Finalising the recipe
☐ Lesson 9: Recipe Presentation
☐ Lesson 10: Debrief
☐ Optional Learning Experiences
☐ Other (Please specify)

If 'Other' please specify which 'other' lesson plans/materials you used.

Student Learning Environment in the Home

What percentage of your students had access to digital devices at home?

◯ 0%
◯ 1-10%
◯ 11-20%
◯ 21-30%
◯ 31-40%
◯ 41-50%
◯ 51-60%
◯ 61-70%
◯ 71-80%
◯ 81-90%
◯ 91-99%
◯ 100%

What platform did you use to deliver the FEAST lessons in the school setting or the home setting?

Please tick as many as apply.

☐ Google slides
☐ Google classroom
☐ FEAST editable PDFs
☐ Zoom
☐ Microsoft Teams
☐ Other (Please specify)

If, 'Other', please specify... what 'other' platform you used to deliver the FEAST lessons in the school setting or the home setting? ____________________

Did you use the FEAST online learning lesson plans OzHarvest provided or did you adapt the lessons?

Please tick as many as apply.

- ☐ Used the FEAST online learning lessons provided by OzHarvest
- ☐ Adapted the lessons provided by OzHarvest
- ☐ A combination of both (used OzHarvest FEAST online learning lessons and adapted them)
- ☐ Other (Please specify)

If 'Other' please specify.... what 'other' lesson plans you used to deliver the FEAST program? __________________________

During school closures, when students were learning from HOME, please rate the effectiveness of the FEAST online learning resources, in aiding you to deliver the FEAST program.

	Did not receive resource	Very effective	Effective	Moderately effective	Slightly effective	Not effective	Did not use this resource
Teacher Instructions	◯	◯	◯	◯	◯	◯	◯
8 Interactive Lessons	◯	◯	◯	◯	◯	◯	◯
Practical Learning	◯	◯	◯	◯	◯	◯	◯
Online individual support from OzHarvest	◯	◯	◯	◯	◯	◯	◯

Practical Components (Food preparation & Cooking activities)

How did you provide the FEAST practical components (i.e. food preparation and cooking activities) to your students?

Please tick as many as apply.

- ☐ In the classroom setting, face-to-face
- ☐ Students did cooking activities at home
- ☐ Other
- ☐ NOT able to do any cooking activities

What 'other' ways did you conduct the practical/cooking components?

e.g. Community kitchen __________________________

How many food preparation/cooking activities did you do with this CLASS in the CLASSROOM (i.e. face-to-face)?

- ◯ 0
- ◯ 1
- ◯ 2
- ◯ 3
- ◯ 4
- ◯ 5
- ◯ 6

How many food preparation/cooking activities did your students do at HOME with their families?

- ◯ 0
- ◯ 1
- ◯ 2
- ◯ 3
- ◯ 4
- ◯ 5
- ◯ 6

How many food preparation/cooking activities did your students do in the 'other' setting(s)?

e.g. Community Kitchen

- ◯ 0
- ◯ 1
- ◯ 2
- ◯ 3
- ◯ 4
- ◯ 5
- ◯ 6

How did your students show you that they cooked at home?

Please tick as many as apply.

- ☐ They photographed the food they cooked and emailed an image.
- ☐ They took a video and emailed the video.
- ☐ They stated they had cooked it.
- ☐ They were not required to show they had cooked it.
- ☐ Other... please specify

If 'Other' please specify...which 'other' way your students showed you that they cooked at home? __________________________________

Which specific FEAST RECIPES did you use?

Please tick as many as apply.

- ☐ Peach Parfait
- ☐ Fruit Skewers with Natural Yoghurt
- ☐ Muesli Bliss Balls
- ☐ Tzatziki Dip with Vegetable Sticks
- ☐ Quick Pickle Vegetables
- ☐ Bircher Muesli with Apples and Bananas
- ☐ Rainbow Salad Roll
- ☐ Sandwich Sushi
- ☐ Chicken & Lentil Kofta Pita Pockets
- ☐ Crunchy Noodle Salad
- ☐ Banana Pikelets
- ☐ Turkish Carrot & Yoghurt Dip
- ☐ Fast Fritters
- ☐ From-the-Fridge Omelette
- ☐ French Toast
- ☐ Wholemeal Burrito Wrap
- ☐ Rainbow Honey Soy Noodle Stir Fry
- ☐ San Choy Bau
- ☐ Tortilla Wraps
- ☐ Butter Bean Hummus
- ☐ Other
- ☐ None of them

If 'other' please specify which other recipe(s) you used.

Student Cooking in the Home

What percentage of your students were able to do SOME cooking at HOME during the SCHOOL CLOSURES?

- ◯ 0
- ◯ 1-10
- ◯ 11-20
- ◯ 21-30
- ◯ 31-40
- ◯ 41-50
- ◯ 51-60
- ◯ 61-70
- ◯ 71-80
- ◯ 81-90
- ◯ 91-99
- ◯ 100

For the students that could NOT cook at home, do you know what barriers restricted their ability to cook at home?

Please tick as many as apply.

- ☐ No, I do not know
- ☐ Lack of access to ingredients
- ☐ Lack of access to kitchen facilities
- ☐ No parental/caregiver supervision
- ☐ Student disengagement
- ☐ Other

If 'Other', please specify...
For the students that could NOT cook at home, do you know what 'other' barriers restricted their ability to cook at home?

____

Did you display the fruit and vegetable posters to your students when they were answering the:

	Yes	No
Pre-FEAST student survey (1st survey)	◯	◯
Post-FEAST student survey (final survey)	◯	◯

During which SCHOOL TERM did the implementation of FEAST take place?

Please pick one only.

- ◯ Term 3 only
- ◯ Term 4 only
- ◯ Both Terms 3 and 4

How has it been for you, running this program during COVID19?

Please tick as many as apply.

- ☐ Difficult
- ☐ Challenging
- ☐ Stressful
- ☐ Manageable
- ☐ Enjoyable
- ☐ Easy
- ☐ Fun
- ☐ Rewarding
- ☐ Other (please specify)

If 'Other', please specify how it has been for you to run FEAST during school closures.

____

Feedback

What could have been done better to help you deliver the FEAST program during the COVID19 school closures?

____

Is there anything else you would like to share about the impact of COVID19 school closures on the FEAST program?

____

 projectredcap.org REDCap
