# Supplementary material for: Impact and process evaluation of a primary-school Food Education and Sustainability Training (FEAST) program in 10-12-year-old children in Australia: pragmatic cluster non-randomized controlled trial
Source: BMC Public Health. 2024 Mar 1;24:657. doi: 10.1186/s12889-024-18079-8 (PMC10905805; doi:10.1186/s12889-024-18079-8)
Supplement: Supplementary file 4 — Additional file 4: Teacher surveys - FEAST lessons plans used by teachers during Implementation (n = 9 teachers, n = 15 class groups) [file 12889_2024_18079_MOESM4_ESM.pdf]

Additional file 4: Teacher surveys - FEAST lessons plans used by teachers during Implementation (n=9 teachers, n=15 class groups)

Lesson #	FEAST LESSON PLANS used by teachers	No. of Classes using each lesson plan	% of Classes using each lesson plan
1	The essential question and scenario	8/15	53.3%
2	Understanding food waste	8/15	53.3%
3	Change-makers and where does food come from?	8/15	53.3%
4	Using STEM Thinking for Problem Solving	2/15	13.3%
5	Understanding food waste	8/15	53.3%
6	Learning about healthy eating	7/15	46.6%
7	Generating Ideas	5/15	33.3%
8	Finalising the recipe	5/15	33.3%
9	Recipe Presentation	3/15	20.0%
10	Debrief	3/15	20.0%
	Optional Learning Experiences	0/15	0.0%
