# Supplementary material for: Impact and process evaluation of a primary-school Food Education and Sustainability Training (FEAST) program in 10-12-year-old children in Australia: pragmatic cluster non-randomized controlled trial
Source: BMC Public Health. 2024 Mar 1;24:657. doi: 10.1186/s12889-024-18079-8 (PMC10905805; doi:10.1186/s12889-024-18079-8)
Supplement: Supplementary file 5 — Additional file 5: Teacher Survey? teacher’s perceptions of student’s learnings during the FEAST program (n = 9 teachers) [file 12889_2024_18079_MOESM5_ESM.pdf]

Additional file 5: Teacher Survey – teacher's perceptions of student's learnings during the FEAST program (n=9 teachers)

	Strongly agree	Agree	Neutral	Disagree	Strongly disagree	NA	DNR
My students found the FEAST activities easy to follow in the classroom.	2	5	0	1	0	0	1
My students found the FEAST activities engaging in the classroom.	3	4	0	1	0	0	1
My students found the FEAST website easy to navigate in the classroom.	2	2	0	1	0	3	1
My students found FEAST online theory lessons easy to do at home during COVID-19 school closures.	3	0	2	1	0	0	1
My students found FEAST cooking activities easy to do at home during COVID-19 school closures.	4	0	1	1	0	2	1
The FEAST program helped students to understand why it is important to be aware of food waste	5	3	0	0	0	0	1
Students are able to understand which behaviours can reduce food waste in the home and at school.	2	6	0	0	0	0	1
Students have increased knowledge and understanding of food waste on a local level.	3	5	0	0	0	0	1
Students have increased knowledge and understanding of food waste on a global level.	2	6	0	0	0	0	1
My students understand where food comes from.	3	5	0	0	0	0	1
My students understand how to make healthier food choices.	3	5	0	0	0	0	1
My students understand how to prepare and cook food.	4	4	0	0	0	0	1
My students understand how to minimise food waste.	2	6	0	0	0	0	1
My students are eating more fruits and vegetables.	3	3	2	0	0	0	1
My students are eating less 'junk' food.	2	2	3	1	0	0	1

Legend: *NA* Not applicable; *DNR* Did not respond
