# Supplementary material for: Impact and process evaluation of a primary-school Food Education and Sustainability Training (FEAST) program in 10-12-year-old children in Australia: pragmatic cluster non-randomized controlled trial
Source: BMC Public Health. 2024 Mar 1;24:657. doi: 10.1186/s12889-024-18079-8 (PMC10905805; doi:10.1186/s12889-024-18079-8)
Supplement: Supplementary file 6 — Additional file 6: Teacher Survey? teacher’s rating FEAST educational content and program delivery (n = 9 teachers) [file 12889_2024_18079_MOESM6_ESM.pdf]

Additional file 6: Teacher Survey – teacher's rating FEAST educational content and program delivery (n=9 teachers)

	Strongly agree	Agree	Neutral	Disagree	Strongly disagree	NA	DNR
The FEAST program content satisfactorily aligned with the identified Year 5 & 6 KLAs	2	6	0	0	0	0	1
The FEAST program satisfactorily aligned with cross-curriculum priority of sustainability	5	3	0	0	0	0	1
I found the STEM lesson plans easy to implement in my classroom	2	3	1	0	0	2	1
The FEAST program satisfactorily aligned with the general capabilities	2	5	1	0	0	0	1
The FEAST program met my student's learning need	4	4	0	0	0	0	1
The FEAST website was easy for me to navigate	3	2	1	2	0	0	1
The FEAST program was easy to integrate face-to-face into my daily classroom routine	4	2	1	1	0	0	1
The FEAST program was easy to teach remotely during COVID-19 school closures	1	2	2	2	1	0	1

Legend: *DNR* Did not respond; *NA* Not Applicable; *KLAs* Key Learning Areas in the Australian Curriculum i.e. English, STEM (Science, Technology, Engineering, and Mathematics), Health and Physical Education; General capabilities (Literacy and Numeracy)
