# Supplementary material for: Impact and process evaluation of a primary-school Food Education and Sustainability Training (FEAST) program in 10-12-year-old children in Australia: pragmatic cluster non-randomized controlled trial
Source: BMC Public Health. 2024 Mar 1;24:657. doi: 10.1186/s12889-024-18079-8 (PMC10905805; doi:10.1186/s12889-024-18079-8)
Supplement: Supplementary file 7 — Additional file 7: Teacher surveys? teacher’s satisfaction with FEAST teacher training courses [file 12889_2024_18079_MOESM7_ESM.pdf]

Additional file 7: Teacher surveys – teacher's satisfaction with FEAST teacher training courses

	Strongly agree	Agree	Neutral	Disagree	Strongly disagree	NA
Q. The FEAST face-to-face training course prepared me to deliver the FEAST program in the following setting (n=3 teachers)						
Classroom setting	3/3					
Home setting		1/3	2/3			
The FEAST online training course prepared me to deliver the FEAST program in the following setting (n=6 teachers)						
Classroom setting	3/6	2/6	1/6			
Home setting	1/6	2/6		2/6		1/6
Did you find the Teacher Professional Development Training course delivered by OzHarvest effective when they delivered it as						
Face-to-face Training	3/3					
Online Training	1/6	5/6				

Legend: NA Not applicable; n=3 teachers undertook face-to-face training; n=6 teachers undertook online training
