# Supplementary material for: Impact and process evaluation of a primary-school Food Education and Sustainability Training (FEAST) program in 10-12-year-old children in Australia: pragmatic cluster non-randomized controlled trial
Source: BMC Public Health. 2024 Mar 1;24:657. doi: 10.1186/s12889-024-18079-8 (PMC10905805; doi:10.1186/s12889-024-18079-8)
Supplement: Supplementary file 8 — Additional file 8: FEAST Student Surveys (Intervention Schools) - Satisfaction of Program N = 261 [file 12889_2024_18079_MOESM8_ESM.pdf]

Additional file 8: FEAST Student Surveys (Intervention Schools) - Satisfaction of Program N=261

FEAST Intervention Student Questions	No. (%) of students responding 'yes'	No. (%) of students responding 'no'	No. (%) of students not responding
Were the FEAST activities easy to read?	232 (88.9)	13 (4.9)	16 (6.1)
Were the FEAST activities easy to understand	223 (85.4)	22 (8.4)	16 (6.1)
Was the FEAST website easy to use?	229 (87.7)	16. (6.1)	16 (6.1)
Was the FEAST program fun?	212 (81.2)	33 (12.6)	16 (6.1)
Were the FEAST lessons easy to do from home?	180 (68.9)	61 (23.4)	20 (7.7)
Did you do the FEAST lessons online?	170 (65.1)	70 (26.8)	21 (8.1)
Did you cook some of the FEAST recipes with your family at home?	131 (50.2)	109 (41.7)	21 (8.1)
Were the FEAST cooking activities easy to do at home?	118 (45.2)	9 (3.5)	133 (50.9)
Did you enjoy cooking with your family?	122(46.7)	7 (2.7)	132 (50.6)
Would you like to continue cooking with your family?	121 (46.4)	8 (3.0)	132 (50.6)
Did you participate in the cooking activities at school with your class?	229 (87.8)	16 (6.1)	16 (6.1)
Did you enjoy cooking in class with your classmates?	217 (83.1)	10 (3.8)	34 (13.1)
Did you learn something about food preparation and cooking that you did not know before?	172 (65.9)	59 (22.6)	30 (11.5)
Did your class create a cookbook?	94 (36.1)	142 (54.4)	25 (9.6)
Did you enjoy creating the cookbook?	79 (30.3)	11 (4.2)	171 (65.5)
Would you like to do the FEAST program again?	192 (73.6)	45 (17.2)	24 (9.2)
Which was your favourite FEAST recipe? French Toast (Most popular)	85 (32.6)	NA	NA
Which was your favourite FEAST recipe? Banana Pikelets	50 (19.2)	NA	NA
Which was your favourite FEAST recipe? Fruit Skewers with Natural Yoghurt	46 (17.6)	NA	NA
Which was your favourite FEAST recipe? Fast Fritters (with vegetables)	37 (14.2)	NA	NA
Which was your favourite FEAST recipe? Crunchy Noodle Salad	34 (13.0)	NA	NA
Which was your favourite FEAST recipe? Chicken & Lentil Kofta Pita Pockets (Least popular recipe)	3 (1.3)	NA	NA
Number of students reporting they did not like any recipes.	54 (20.1)	NA	NA
