# Supplementary material for: Impact and process evaluation of a primary-school Food Education and Sustainability Training (FEAST) program in 10-12-year-old children in Australia: pragmatic cluster non-randomized controlled trial
Source: BMC Public Health. 2024 Mar 1;24:657. doi: 10.1186/s12889-024-18079-8 (PMC10905805; doi:10.1186/s12889-024-18079-8)
Supplement: Supplementary file 9 — Additional file 9: Teacher Survey? Teacher satisfaction of FEAST resources during COVID-19 school-closures n = 9 [file 12889_2024_18079_MOESM9_ESM.pdf]

Additional file 9: Teacher Survey – Teacher satisfaction of FEAST resources during COVID-19 school-closures n=9

Resources	Very effective	Effective	Moderately effective	Slightly effective	Not effective	Did not use this resource	DNR
During school closures, when students were learning from HOME, please rate the effectiveness of the FEAST online learning resources, in aiding you to deliver the FEAST program.							
Teacher Instructions	1	1		2		1	4
8 Interactive Lessons		2				3	4
Practical Learning	1	1	1			2	4
Online individual support from OzHarvest		1				4	4

Legend: DNR Did not respond
