# Supplementary material for: Impact and process evaluation of a primary-school Food Education and Sustainability Training (FEAST) program in 10-12-year-old children in Australia: pragmatic cluster non-randomized controlled trial
Source: BMC Public Health. 2024 Mar 1;24:657. doi: 10.1186/s12889-024-18079-8 (PMC10905805; doi:10.1186/s12889-024-18079-8)
Supplement: Supplementary file 10 — Additional file 10: Teacher Survey? Teacher satisfaction of FEAST Resources (n = 9 teachers) [file 12889_2024_18079_MOESM10_ESM.pdf]

Additional file 10: Teacher Survey – Teacher satisfaction of FEAST Resources (n=9 teachers)

Resources	Very effective	Effective	Moderately effective	Slightly effective	Not effective	Did not use this resource	Did not receive resource
Please rate the effectiveness of the FEAST resources provided, in aiding you to deliver the FEAST program.							
Online teacher portal	3	2	1	2	0	1	NA
Online student portal	1	2	0	0	0	6	NA
Student resources	3	3	1	1	0	1	NA
FEAST unit of work	3	4	1	1	0	0	NA
STEM lesson plans	3	3	0	1	0	2	NA
FEAST practical guide	4	5	0	0	0	0	NA
Kitchen Kit	7	1	0	0	0	1	NA
Cold recipe book	6	3	0	0	0	0	NA
Hot recipe book	6	3	0	0	0	0	NA
Optional learning experiences	2	3	0	0	0	4	NA

Legend: NA Not applicable
