# Supplementary material for: Impact and process evaluation of a primary-school Food Education and Sustainability Training (FEAST) program in 10-12-year-old children in Australia: pragmatic cluster non-randomized controlled trial
Source: BMC Public Health. 2024 Mar 1;24:657. doi: 10.1186/s12889-024-18079-8 (PMC10905805; doi:10.1186/s12889-024-18079-8)
Supplement: Supplementary file 11 — Additional file 11: FEAST Student Survey (Intervention Schools)? Skills learnt during food preparation and cooking activities (n = 172) [file 12889_2024_18079_MOESM11_ESM.pdf]

Additional file 11 : FEAST Student Survey (Intervention Schools) – Skills learnt during food preparation and cooking activities (n=172)

Skills or Knowledge learnt during food preparation and cooking activities	No. of students	% of students
How to make a specific recipe (Most popular)	36/172	20.93%
How to cook	27/172	15.7%
About food waste	25/172	14.5%
How to measure/cut/dice/mix	13/172	7.6%
How to use utensils/cooking equipment	10/172	5.8%
How to cook with friends	7/172	4.1%
How to cook with imperfect foods	6/172	3.5%
Miscellaneous	27/172	15.7%

Legend: Of the 261 FEAST intervention students that responded to the post-FEAST survey 172 (65.95%) replied ‘yes’ they learnt a new skill
