# Supplementary material for: Impact and process evaluation of a primary-school Food Education and Sustainability Training (FEAST) program in 10-12-year-old children in Australia: pragmatic cluster non-randomized controlled trial
Source: BMC Public Health. 2024 Mar 1;24:657. doi: 10.1186/s12889-024-18079-8 (PMC10905805; doi:10.1186/s12889-024-18079-8)
Supplement: Supplementary file 12 — Additional file 12: Teacher Survey? reports of other nutrition and/or sustainability programmes implemented in intervention schools (n10 Schools) [file 12889_2024_18079_MOESM12_ESM.pdf]

Additional file 12: Teacher Survey – reports of other nutrition and/or sustainability programmes implemented in intervention schools (n=10 Schools)

Program	School 1	School 4	School 7	School 8	School 12	School 13	School 18	School 3	School 10	School 11
Crunch&Sip ®	✓			✓	✓	✓	✓	DNR	WD	WD
Kitchen Garden	✓	✓	✓	✓	✓	✓	✓	DNR	WD	WD
Composting		✓		✓	✓	✓		DNR	WD	WD
Sustainability/environment student group	✓			✓		✓	✓	DNR	WD	WD
Community - farm visits						✓		DNR	WD	WD
Fruit and Vegetable stalls						✓		DNR	WD	WD
Emergency food programmes - e.g. breakfast club	✓	✓				✓		DNR	WD	WD
Healthy canteen policy					✓	✓		DNR	WD	WD
Sustainability policy	✓					✓	✓	DNR	WD	WD
Other (Native bees, tree planting)		✓						DNR	WD	WD
**Totals**	**5/9**	**3/9+**	**1/9**	**4/9**	**4/9**	**9/9**	**4/9**			

Legend: *Crunch&Sip ®* is a set time in primary schools in two Australian states (i.e. New South Wales and Western Australia), for students to 'refuel' on vegetables, salad and/or fruit and 'rehydrate' with water (schools choose whether they want to implement it) ; *DNR* Did not respond; *WD* Schools withdrew after baseline data collection and teachers were not available for post-intervention surveys; + teachers responding that they have other nutrition and/or sustainability programmes in their school, other than those listed on survey list.
